# Metabolic and Redox‐Related Effects of *Mauritia flexuosa* (Buriti) Oil Supplementation in Wistar Rats

**DOI:** 10.1111/1750-3841.71065

**Published:** 2026-04-14

**Authors:** Letícia de Almeida Sant’ Anna Trindade, Beatriz Alem Nascimento de Araújo, Gabriel de Alcantara Noblat, Karen Pereira Coutinho, Karla de Araújo Coutinho, Renata Nascimento Matoso Souto, Anderson Junger Teodoro, Aline D'Avila Pereira, Mariana Sarto Figueiredo

**Affiliations:** ^1^ PPGCM – Graduate Program in Medical Sciences UFF – Fluminense Federal University Niterói Brazil; ^2^ Graduate in Nutrition Faculty UFF – Fluminense Federal University Niterói Brazil; ^3^ Department of Gastronomy, Josué de Castro Nutrition Institute UFRJ – Federal University of Rio de Janeiro Rio de Janeiro Brazil; ^4^ Department of Nutrition and Dietetics, Faculty of Nutrition UFF – Fluminense Federal University Rio de Janeiro Brazil; ^5^ Department of Geography, Institute of Geography UERJ – Rio de Janeiro State University Cabo Frio Brazil

**Keywords:** *Mauritia flexuosa*, buriti oil, body composition, lipid profile, bone parameters, redox balance

## Abstract

*Mauritia flexuosa* (buriti) oil is rich in oleic and palmitic fatty acids, bioactive compounds associated with inflammation regulation, redox balance, and metabolism. This study evaluated the effects of buriti oil supplementation on body and bone composition, biochemical parameters, and redox balance in male Wistar rats. Thirty rats were assigned to the following groups: saline (SC, *n* = 10); soybean oil (SO, *n* = 10); and buriti oil (BURI, *n* = 10). Oils were administered by gavage at a dose of 0.5 mL/100 g body mass for 30 consecutive days. At PN120 (120 days old), body, biochemical, bone, and redox balance parameters were analyzed. BURI group showed lower body mass (−7.6%, *p* < 0.05), serum carbonylated protein (−20.6%, *p* < 0.05), triglycerides (−33.3%, *p* < 0.05), high‐density lipoprotein (HDL, −10.4%, *p* < 0.05), very‐low‐density lipoprotein (VLDL, −33.3%, *p* < 0.05), total proteins (−8.4%, *p* < 0.05), alanine aminotransferase (−10.4%, *p* < 0.05), alkaline phosphatase (−34%, *p* < 0.05), calcium (−7.9%, *p* < 0.05), iron (−32.7%, *p* < 0.05), magnesium (−21.3%, *p* < 0.05), and uric acid (−30.9%, *p* < 0.05), as well as reduced femur width (−16.5%, *p* < 0.05). Conversely, BURI exhibited higher hepatic FRAP activity (+12.9%, *p* < 0.05) and increased femur length (+3.4%, *p* < 0.05). SO group presented lower serum carbonylated protein (−22.7%, *p* < 0.05), triglycerides and VLDL (−37.1% and −37.1%, respectively, *p* < 0.05), total protein (−7.2%, *p* < 0.05), alkaline phosphatase (−26.8%, *p* < 0.05), calcium (−7.7%, *p* < 0.05), iron (−22.2%, *p* < 0.05), magnesium (−14.6%, *p* < 0.05), uric acid (−20.9%, *p* < 0.05), and femur weight and width (−8% and −20.2%, respectively, *p* < 0.05), as well as higher FRAP levels (+8.6%, *p* < 0.05). No changes were observed in body composition among groups. Buriti oil supplementation modulated metabolic pathways, leading to reduced body mass and improved lipid profile, hepatic markers, redox balance, and bone parameters.

**Practical Applications:**

Buriti oil, a native Amazonian fruit oil, improved lipid profile and antioxidant balance in healthy rats, suggesting its potential for functional foods and supplements with antioxidant and anti‐inflammatory benefits.

## 1 Introduction

*Mauritia flexuosa* L.f. (*Arecaceae*) is a hyperdominant dioecious neotropical palm in the Amazon, typical of humid areas, with seeds that germinate in waterlogged soils. Known as buriti, moriti, or aguaje, it has a single stem, a crown‐shaped canopy, and costapalmate leaves. Its fruit is an oval drupe with brown scales and an orange, oily pulp (Delgado et al. 2007; Martins et al. 2012; Koolen et al. 2018; de Ávila et al. 2023).

Buriti oil is rich in carotenoids and tocopherols, especially β‐carotene and α‐tocopherol, with antioxidant and anti‐inflammatory action. Beta‐carotene stands out for its role in protecting against oxidative damage, modulating immune function, and serving as a precursor of vitamin A (Marcelino et al. 2022; Barboza et al. 2022; Silva et al. 2022). Extracted from the pulp and peel, the oil is mainly composed of oleic acid, fourfold than other saturated fatty acids, which reinforces the oxidative stability and functional profile (Aquino et al. 2016; Lozano‐Garzón et al. 2023). Its lower polyunsaturated content contributes to this stability and is higher than oils such as canola and olive (Ferreira et al. 2024).

It contains phytosterols such as campesterol, stigmasterol, and β‐sitosterol, with anti‐atherosclerotic, antitumor, and antioxidant effects (Uddin et al. 2018; Ramos‐Escudero et al. 2022). Tocotrienols, tocopherols, and carotenoids are concentrated in crude oils (Aquino et al. 2015). *Mauritia flexuosa* oil has high levels of β‐carotene, with studies reporting approximately 787 mg/kg in the pulp oil (De Souza Aquino et al. 2023), which explains its intense color and strong antioxidant activity. Marcelino et al. (2022) also highlight buriti as one of the main natural sources of carotenoids. According to the review by Saini et al. (2022), dietary carotenoids, including β‐carotene, have been associated with the modulation of oxidative stress and potential protective effects in chronic diseases. Thus, the concentration of this compound in buriti oil reinforces the interest in investigating its biological effects (Ferreira et al. 2024).

Considered one of the most abundant palms in Brazil, *M. flexuosa* has high nutritional and economic value (Barboza et al. 2022). Its oil is used in the cosmetic industry for its moisturizing, photoprotective, and antimicrobial properties, with applications in cosmetics, aromatherapy, and perfumery (G. S. Pereira et al. 2018; Ibiapina et al. 2022; Pontes et al. 2024). In the food sector, it stands out for its vitamin content, oleic acid, and antioxidants, making it promising for margarines, frying, and heat foods (da Silva et al. 2024). The pulp is used in ice creams, sweets, natural dyes, and fermented drinks (G. S. Pereira et al. 2018; Embrapa 2021).

Despite its nutritional and functional qualities, its industrial use is still limited, mainly concentrated in artisanal consumption in juices, jams, ice creams, and wines (Nascimento‐Silva et al. 2020; Ramos‐Escudero et al. 2022; da Silva et al. 2024). Therefore, this study aimed to investigate the effects of buriti oil supplementation in healthy male Wistar rats, focusing on its impact on lipid, biochemical, oxidative, and anthropometric parameters, as well as on metabolic safety and biological responses under controlled experimental conditions. We hypothesized that buriti oil supplementation would confer metabolic and oxidative benefits even in healthy animals, while presenting low toxicity.

## 2 Materials and Methods

### 2.1 Experimental Methods

The experiment was conducted in the Animal Facility of the Experimental Nutrition Laboratory of the Emília de Jesus Ferreiro School of Nutrition at the Federal University of Fluminense (LABNE/UFF), using male rats of the species *Rattus norvegicus*, Albinus strain, Wistar lineage, provided by the Laboratory Animal Center (NAL/UFF).

### 2.2 Ethical Approval

All procedures involving animals were approved by the Ethics Committee on Animal Use of the Fluminense Federal University (CEUA/UFF, protocol no. 9204110520) and followed the CONCEA guidelines (Law 11.794/2008), the ARRIVE 2.0 recommendations (Percie Du Sert et al. 2020), and the 3Rs principles (Reduction, Refinement, and Replacement). Wistar rats (*R. norvegicus*) were selected due to their docile behavior, ease of handling, small size, rapid reproduction, adaptability, and genetic and biological similarity to humans.

Brazilian legislation (Regulatory Norm N57/2022) requires environmental enrichment. However, commonly used materials (polypropylene toys, tree bark, coconut fiber, paper, wood) can be ingested and alter food intake, potentially affecting outcomes related to weight, biochemical parameters, and bone function. Therefore, enrichment was not used to avoid data bias.

The experimental protocol describes all procedures and establishes euthanasia as the humane endpoint. The intervention involved a low‐dose edible supplement considered noninvasive and compatible with gastric capacity, without compromising animal well‐being. Euthanasia was performed under anesthesia at the end of the study, as approved by CEUA/UFF, respecting pain and stress reduction measures. Any unexpected adverse effect would lead to immediate intervention, including analgesia or humane euthanasia under anesthesia. Therefore, to minimize stress and suffering, according to the project's invasiveness level, which would be Grade I—Mild, the animals were frequently monitored for early signs of pain and stress; all users involved in animal care will be properly trained and guided by a veterinarian. The animals were not to be restrained for prolonged periods, respecting the physiological and behavioral needs of the species (according to RN 30/ 2016 CONCEA/MCTI and the Brazilian Guide for the Production, Maintenance, or Use of Animals in Teaching or Scientific Research Activities [CONCEA 2023]).

### 2.3 Experimental Design

Thirty newly weaned male Wistar rats (*R. norvegicus*, Albinus), between 21 and 30 days old, were used. At 90 days of age (PN90), the animals were randomly divided into three groups (*n* = 30): (1) control saline group, receiving a commercial diet and gavage with saline solution (SC, *n* = 10); (2) soybean oil group, receiving a commercial diet and gavage with soybean oil (SO, *n* = 10); and (3) buriti oil group, receiving a commercial diet and gavage with buriti oil (BURI, *n* = 10). No animals were excluded after the experiment started. During analysis, no a priori criteria were established for excluding data points, and all collected data were included in the statistical evaluations. The animals were kept in collective cages (three to four animals per cage), measuring 26.5 cm high × 34.1 cm wide × 49.7 cm deep. The cages were fixed to shelves with microisolators, with individual ventilation and exhaust systems. Housing conditions were as follows: a 12‐h light–dark cycle, controlled temperature (21°C–23°C), and humidity (60 ± 10%). Sample size was calculated based on a mean body weight of 200 g with a standard deviation of 25 g, using an alpha level of 0.05. This resulted in 10 animals per group, with a sampling error of 7% (Miot 2011). All treatments and measurements were conducted by at least two researchers to reduce confounding factors and minimize errors in data collection. The animals were kept in the Animal Facility of the Faculty of Nutrition/UFF.

At PN120, the animals were anesthetized (Ketamine 90 mg/kg + Xylazine 10 mg/kg) and euthanized for blood collection by cardiac puncture, as well as for the removal of the femurs, liver, pancreas, adrenal glands, and white (retroperitoneal, mesenteric, epididymal, and subcutaneous) and brown adipose tissues. Blood was centrifuged (15 min, 1600 × *g*, 4°C) to obtain the serum and stored at −20°C. Tissues were weighed and stored at −80°C for later analyses. All biological samples from animal models were stored in the Faculty of Nutrition biorepository, and all data collected from the analyses were saved in protocol brochures and on computer drives. No adverse events, expected or unexpected, were observed during the experiment.

### 2.4 Diet and Gavage

During the experiment, all animals received a commercial diet (Nuvilab, NUVITAL, PR, Brazil) containing 21.49% of protein, 69.65% of carbohydrate, and 8.83% of lipid until PN180, and filtered water ad libitum.

The animals received gavage with soybean oil or buriti oil for 30 consecutive days at a dosage of 0.5 mL/100 g of body mass, and gavage with saline solution was administered in the same proportion as the oils (Raghu Nadhanan et al. 2013; CONCEA 2016). Soybean oil (Liza) was purchased from a local store. Buriti oil was obtained from Cooperativa Grande Sertão (Minas Gerais, Brazil), which extracted the oil by cold mechanical pressing. Vegetal oil doses supplemented were based on the study by Raghu Nadhanan et al. (2013), which supplemented fish oil to young adult rats at a dosage of 0.5 mL/100 g of body mass. Soybean oil was used as a second control group, since soybean oil is recommended by the American Institute of Nutrition (AIN) as a source of lipids for rodents (Reeves et al. 1993).

### 2.5 Fatty Acid Profile in Soybean Oil and Buriti Oil

Lipid extraction, saponification, and methylation of fatty acids from vegetable oils were performed according to the method previously described by Souto et al. (2025).

The fatty acid composition of soybean and buriti oils was determined in triplicate by capillary gas chromatography (GC) after conversion of total lipids into fatty acid methyl esters (FAMEs) through direct transesterification. An aliquot of 1.0 µL of the derivatized sample, diluted in *n*‐hexane, was injected into a gas chromatograph (Shimadzu GC‐2014, Kyoto, Japan) fitted with a flame ionization detector (FID) and a split/splitless injector operating at a 1:20 split ratio. Separations were carried out using an Omegawax‐320 capillary column (30 m × 0.25 mm internal diameter, 0.25 µm film thickness; Supelco, Bellefonte, PA, USA). The oven temperature program began at 170°C, held for 3 min, followed by a gradual increase of 1°C per minute to 225°C, where it was maintained for an additional 5 min. Helium served as the carrier gas under a constant pressure of 100 kPa. The injector and detector temperatures were set at 260°C and 280°C, respectively. Identification of FAMEs was achieved by comparing their retention times with those of a commercial standard mixture. Quantification was conducted by internal normalization of chromatographic peak areas, applying theoretical response factors, and results were expressed as g/100 g of total fatty acids. Fatty acid quantification was performed via internal normalization of peak areas, adjusted using theoretical correction factors, and expressed as g/100 g of total fatty acids (Souto et al. 2025) (Table 3).

### 2.6 Body Mass, Nasoanal Length, Lee Index, and Body Mass Index

Body weight was monitored daily, at the same time, using a digital scale (Filizola MF‐3, 0.5 g) to track weight gain and calculate oil doses. Food intake was measured weekly throughout the experiment.

At PN120, the nasoanal length (cm, distance from nose tip to anus), Lee index (^3^√body mass [g] / nasoanal length [cm]), and body mass index (BMI, body weight [g] / nasoanal length squared [cm^2^]) were determined (Novelli et al. 2007; da Silva Nery et al. 2011). These analyses were conducted at the Experimental Nutrition Laboratory of the Fluminense Federal University (LABNE).

### 2.7 Dual‐Energy X‐Ray Absorptiometry (DXA)—Body Composition

Body composition was assessed before (PN90) and after treatment (PN120) by DXA, using the GE Lunar iDXA 200,368 device (Lunar, Wisconsin, USA) and BIS 2008 software (version 12.20, GE Healthcare). The following parameters were measured: lean mass (g), fat mass (g), body fat percentage (%), bone mineral density (g/cm^2^), bone mineral content (g), and bone area (cm^2^). The analyses were conducted at the Nutritional and Functional Assessment Laboratory of the Fluminense Federal University (LANUFF).

### 2.8 Fasting Blood Glucose and Biochemical Profile

All subsequent analyses were performed using 10 animals per experimental group (*n* = 10).

At PN120, fasting blood glucose was measured using a glucometer (ACCUCHEK Advantage) from tail vein blood. Serum was used for biochemical analyses in an automated analyzer (Bioclin BST20, Quibasa—MG), including albumin (g/dL), total protein (g/dL), total cholesterol (mg/dL), HDL‐c (mg/dL), LDL‐c (mg/dL), triglycerides (mg/dL), ALT (mg/dL), AST (mg/dL), total and direct bilirubins (mg/dL), calcium (mg/dL), phosphorus (g/dL), iron (µg/dL), magnesium (g/dL), alkaline phosphatase (mg/dL), urea (mg/dL), and uric acid (mg/dL), all measured by colorimetric methods with Bioclin kits. VLDL‐c was calculated by dividing triglycerides by 5, and LDL‐c was calculated by using the following formula: LDL‐c = total cholesterol − (HDL‐c + VLDL‐c). For analysis, samples were thawed, vortexed, distributed in cuvettes with reagents, and processed in the automatic equipment. Biochemical analyses were conducted at the Experimental Nutrition Laboratory of the Fluminense Federal University (LABNE).

Serum insulin was measured by radioimmunoassay (ng/mL) at the Obesity and Comorbidities Research Center of the State University of Campinas (OCRC), and the insulin resistance index (IRI) was calculated by multiplying glucose (mmol/L) by insulin (μIU/mL).

### 2.9 Serum and Hepatic Redox Balance

All of the following analyses were performed at the Experimental Nutrition Laboratory (LABNE) and at the Integrated Food and Nutrition Center of the Faculty of Nutrition (UFF).

All subsequent analyses were described in detail above, and all experiments were performed using 10 animals per experimental group (*n* = 10).

#### 2.9.1 DPPH Radical Scavenging Activity

DPPH radical scavenging activity in serum and liver samples was evaluated according to Brand‐Williams et al. (1995), with adaptations for hepatic tissue. Samples were incubated with DPPH in a methanolic solution for 30 min and protected from light. Radical reduction was measured at 515 nm using a microplate reader (SpectraMax i3x MultiMode, USA). Analyses were performed in triplicate. Quantification was based on a Trolox standard curve, with results expressed in micromoles of Trolox equivalents (TE) per gram.

#### 2.9.2 Total Antioxidant Activity by Ferric Reducing Antioxidant Power (FRAP)

As described by Rufino et al. (2006), the method evaluates antioxidant capacity by converting the Fe^3^^+^–TPTZ complex (light blue) to Fe^2^^+^–TPTZ (dark blue), mediated by antioxidants in an acidic medium. Serum and liver homogenates were incubated with FRAP reagent for 30 min at 37°C and protected from light. Absorbance was read at 595 nm using a microplate reader. Quantification was based on a ferrous sulfate standard curve with four dilutions for serum and seven for tissue, expressed in micromoles of ferrous sulfate per milligram of sample protein.

#### 2.9.3 . Thiobarbituric Acid Reactive Substances (TBARS)

Lipid peroxidation was assessed by quantifying TBARS, as per Feldman (2004), with modifications for liver homogenate. Malondialdehyde (MDA), a lipid peroxidation byproduct, reacts with TBA, forming a pink compound measurable by spectrophotometry. For serum, 100 µL was mixed with 100 µL of KPE buffer. For tissue, 200 µL of homogenate was mixed with 350 µL of 10% TCA and cooled (15 min at 4°C for serum, 20 min for tissue). After centrifugation (15 min, 2200 × *g*), 300 µL of the supernatant was added to 300 µL of TBA and incubated at 95°C for 1 h. Solutions were cooled for 15 min at room temperature, and 150 µL was transferred to ELISA plates and read at 532 nm. Results were calculated using a standard curve and expressed in nanomoles of TBARS per milliliter.

#### 2.9.4 Protein Carbonyl Content

Protein oxidation in serum and liver homogenates was assessed based on Mesquita et al. (2014), with tissue adaptations, by quantifying carbonyl derivatives after reaction with 2,4‐dinitrophenylhydrazine (DNPH) (10 mM in 0.5 M H_3_PO_4_) in alkaline medium. For serum, 320 µL was mixed with 160 µL of DNPH; for tissue, 160 µL of the sample was mixed with 320 µL of DNPH. Samples were vortexed, incubated for 10 min at room temperature, and protected from light. Controls received only H_3_PO_4_. After incubation, 80 µL of NaOH (6 M) was added, followed by centrifugation (4000 rpm, 5 min, 25°C). The mixture was incubated for another 5 min before reading at 450 nm in ELISA plates. Reaction stability required 10 min of incubation with NaOH. Carbonyl content was calculated using a molar extinction coefficient of 22,308 M^−^^1^·cm^−^^1^ and expressed in micromoles per milligram of protein.

#### 2.9.5 Thiol

Total reduced thiols were quantified using Ellman's reagent (DTNB, 5,5'‐dithiobis‐(2‐nitrobenzoic acid)) as described by Ellman (1959). For the reaction, 20 µL of serum or liver homogenate was mixed with 160 µL of thiol buffer (Tris 200 mM and EDTA 20 mM, pH 8.2), 820 µL of absolute methanol, and 10 µL of DTNB 10 mM (3.9 mg/mL in methanol). The mixture was vortexed, incubated for 15 min at room temperature, and centrifuged (3000 × *g*, 15 min, 25°C). Absorbance was read at 412 nm in ELISA plates. The reaction releases TNB (5‐thio‐2‐nitrobenzoate), which was quantified using the molar extinction coefficient of 4.46 ± 0.23 × 10^4^ M^−^^1^·cm^−^^1^, with results expressed in nanomoles of reduced DTNB per milligram of sample protein. Under oxidative stress, DTNB conversion to TNB is reduced. To correct for interference, control samples (without DTNB) were used to subtract absorbance from heme‐containing substances (Frankenfeld et al. 2014).

#### 2.9.6 Ferrous Oxidation in Xylenol Orange (FOX)

Lipid hydroperoxides were quantified by the FOX method (Wolff 1994), based on the oxidation of Fe^2+^ to Fe^3+^ in the presence of hydroperoxides, forming a complex with xylenol orange measurable at 560 nm. This is a sensitive method to detect low levels of hydroperoxides. For the analysis, 90 µL of the sample was mixed with 10 µL of 40 mM BHT (45 mg in 5 mL methanol) and vortexed for 1 min. After centrifugation (5000 × *g*, 5 min, 4°C), 100 µL of supernatant was added to 900 µL of reagent (90% methanol, 100 µM xylenol orange, 25 mM H_2_SO_4_, and 250 µM ferrous sulfate). The mixture was incubated for 2 h at room temperature in the dark and centrifuged (10,000 × *g*, 10 min, 4°C). Absorbance was measured at 560 nm. Results, expressed in micromoles per liter, were calculated using a molar extinction coefficient of 4.46 ± 0.23 × 10 M^−^^1^·cm^−^^1^.

### 2.10 Radiodensity of the Left Femoral Head

At PN120, the radiodensity of the clean left femoral head was measured. The analysis was performed using a tomography scanner (Brilliance 64, Philips) at the Antonio Pedro University Hospital of the Fluminense Federal University (HUAP/UFF), with the help of a laboratory technician to handle the device. The femurs were placed on a flat surface on the device's conveyor belt for imaging. The images were obtained through axial sections of the bone, 1 mm thick. The reading of the bone images obtained in the tomography was performed using the Phillips DICOM Viewer program, using the Tool‐Elipse measurement, and the radiodensity result found was the average value of three readings, expressed in Hounsfield units (HU) (da Costa et al. 2011).

### 2.11 Biomechanics of the Left Femur

Biomechanical properties assessed included maximum and breaking force (N), elastic modulus, and maximum and breaking stress (MPa), using a three‐point bending test in a Universal Testing Machine (EMIC, DL 2000) at the Analytical Laboratory of Restorative Biomaterials at the Fluminense Federal University (LABIOM/UFF). A 200‐kgf perpendicular force was applied to the bone's medial region at 0.5 mm/min until fracture. Femurs were supported on 3‐mm‐diameter rollers, with a 21.70‐mm radius (Latempa et al. 2014).

### 2.12 Femur Anatomical Parameters

Femur dimensions were measured using a digital caliper (MTX, 150 mm) and an analytical scale (Edutec STR 224) to determine mass (g) and length (mm) between epiphyses. From these data, the midpoint, diaphysis width, and distances between the greater and lesser trochanters and between the lateral and medial epicondyles were calculated (da Costa et al. 2011), with the analysis conducted at the Experimental Nutrition Laboratory of the Fluminense Federal University (LABNE).

### 2.13 DXA of the Left Femur

DXA was performed on the left femur to assess bone content. A container filled with raw rice was used to differentiate soft tissue mass. Measurements included bone mineral density (BMD, g/cm^2^), bone mineral content (BMC, g), and bone area (cm^2^) (da Costa et al. 2011), carried out at the Nutritional Assessment Laboratory of the Faculty of Nutrition (UFF).

### 2.14 Statistical Analysis

Statistical analyses were performed using GraphPad Prism version 9.0. Normality was assessed using the Shapiro–Wilk test. Results are expressed as mean ± standard error of the mean (SEM) or as median and interquartile range (IQR), depending on data distribution. One‐way ANOVA was used for group comparisons, followed by Tukey's post hoc test when appropriate. A *p*‐value of <0.05 was considered statistically significant.

## 3 Results

**FIGURE 1:**
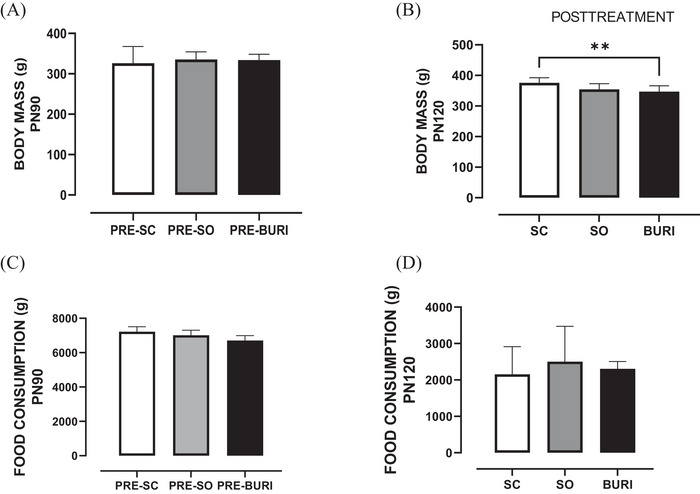
Body mass and food intake before and after supplementation with soybean and buriti oils. (A) Body mass gain (g) from PN25 to PN90 before supplementation. (B) Body mass gain (g) from PN90 to PN120 after supplementation. (C) Total food intake (g) from PN25 to PN90 before supplementation. (D) Total food intake after supplementation (g) from PN90 to PN120. PRE‐SC (*n* = 10): group receiving a commercial diet before saline gavage; PRE‐SO (*n* = 10): group receiving a commercial diet before soybean oil gavage; PRE‐BURI (*n* = 10): group receiving a commercial diet before buriti oil gavage; SC: saline control group receiving a commercial diet and gavage with saline (*n* = 10); SO: soybean oil group receiving a commercial diet and gavage with soybean oil (dose: 0.5 mL/100 g body mass) (*n* = 10); BURI: buriti oil group receiving a commercial diet and gavage with buriti oil (dose: 0.5 mL/100 g body mass) (*n* = 10); PN25: 25 days old; PN90: 90 days old; PN120: 120 days old. Results expressed as mean and standard error of the mean. Symbol ** indicates statistical difference between the BURI and SC groups (*p* < 0.05) (B). No symbol indicates no statistical difference among the groups (*p* > 0.05) (A, C, and D).

At PN90, no significant differences were observed in body weight (Figure 1A) and food intake (Figure 1C) among groups (*p* > 0.05). At PN120, the BURI group showed a −7.6% reduction in body weight compared to the SC group (*p* < 0.05; Figure 1B). There was no significant difference in food intake among the groups after treatment (*p* > 0.05; Figure 1D). The results related to body weight and food intake of the animals before and after supplementation are shown in Figure 1.

In the same direction, at PN90, there were no significant differences in body fat percentage (Figure 2A), body fat (g) (Figure 2C), lean mass (g) (Figure 2E), bone mineral density (g/cm^2^) (Figure 2G), bone mineral content (g) (Figure 2I), and bone area (cm^2^) (Figure 2K) between groups (*p* > 0.05). At PN120, there were no significant differences between groups in body fat percentage (Figure 2B), body fat (g) (Figure 2D), lean mass (g) (Figure 2F), bone mineral density (g/cm^2^) (Figure 2H), bone mineral content (g) (Figure 2J), and bone area (cm^2^) (Figure 2L) (*p* > 0.05). The results related to the body composition of the animals before and after supplementation are shown in Figure 2.

FIGURE 2Body composition of animals before and after supplementation with soybean and buriti oils. (A) Body fat (%) at PN90 before supplementation. (B) Body fat (%) at PN120 after supplementation. (C) Body fat mass (g) at PN90 before supplementation. (D) Body fat mass (g) at PN120 after supplementation. (E) Total lean mass (g) at PN90 before supplementation. (F) Total lean mass (g) at PN120 after supplementation. (G) Bone mineral density (g/cm^2^) at PN90 before supplementation. (H) Bone mineral density (g/cm^2^) at PN120 after supplementation. (I) Bone mineral content (g) at PN90 before supplementation. (J) Bone mineral content (g) at PN120 after supplementation. (K) Bone area (cm^2^) at PN90 before supplementation. (L) Bone area (cm^2^) at PN120 after supplementation. PRE‐SC (*n* = 10): group receiving a commercial diet before saline gavage; PRE‐SO (*n* = 10): group receiving a commercial diet before soybean oil gavage; PRE‐BURI (*n* = 10): group receiving a commercial diet before buriti oil gavage; SC: saline control group receiving a commercial diet and gavage with saline (*n* = 10); SO: soybean oil group receiving a commercial diet and gavage with soybean oil (dose: 0.5 mL/100 g body mass) (*n* = 10); BURI: buriti oil group receiving a commercial diet and gavage with buriti oil (dose: 0.5 mL/100 g body mass) (*n* = 10); PN25: 25 days old; PN90: 90 days old; PN120: 120 days old. Results expressed as mean and standard error of the mean. No symbol in figures indicates no statistical difference among the groups (*p* > 0.05).

**Figure:**
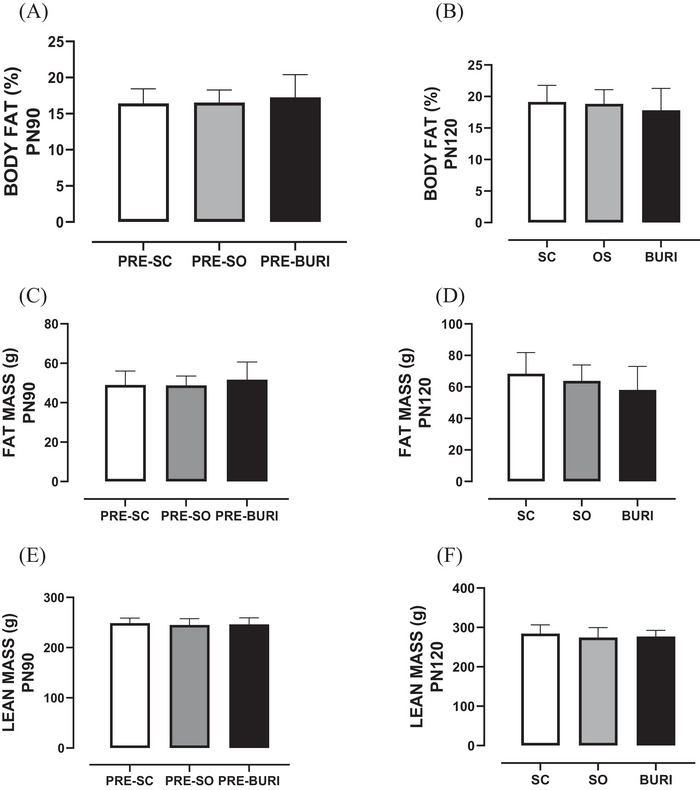

**Figure:**
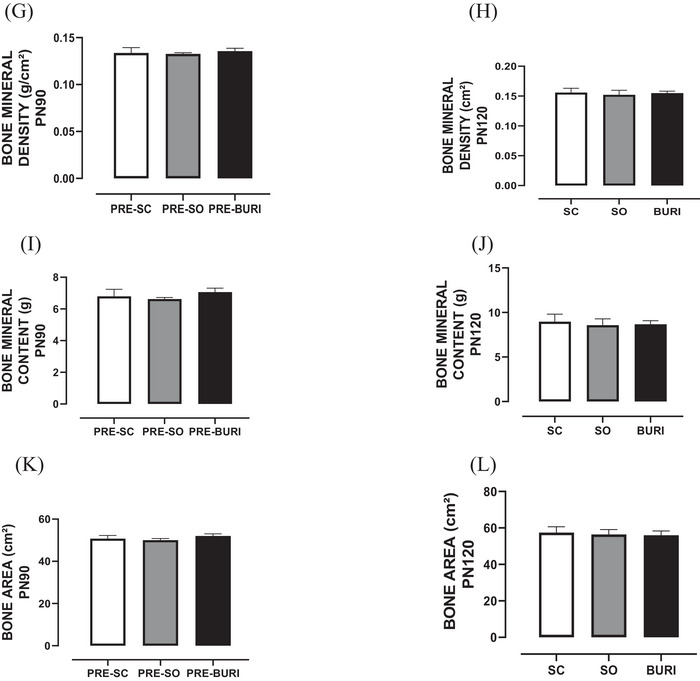

**FIGURE 3:**
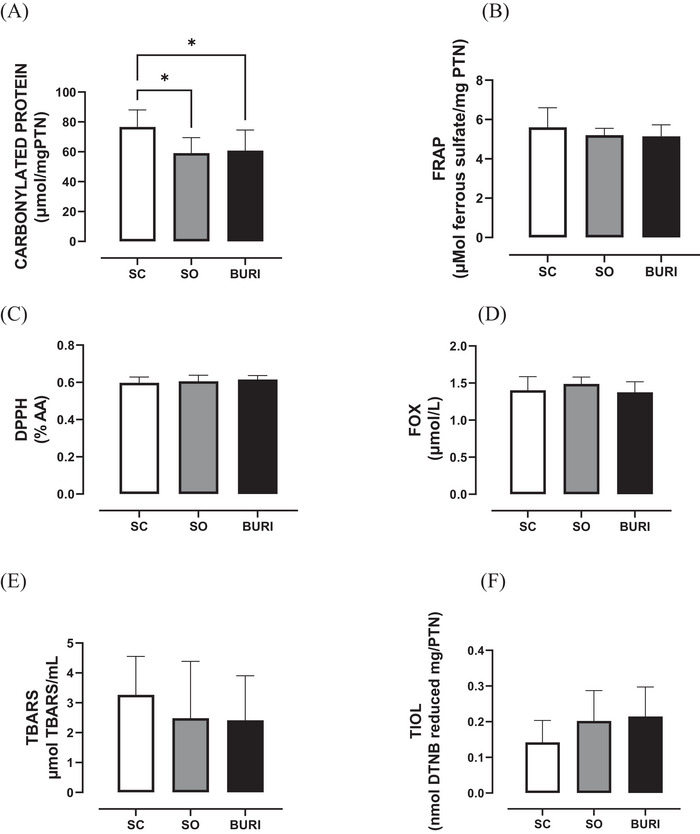
Serum redox balance at PN120. (A) Serum carbonylated protein (µmol mg/PTN) at PN120. (B) Serum FRAP (µmol ferrous sulfate/mg protein) at PN120. (C) Serum DPPH (mM DPPH/mg protein) at PN120. (D) Serum FOX (µmol/L) at PN120. (E) Serum TBARS (µmol TMP/mL) at PN120. (F) Thiol (nmol reduced DTNB/mg protein) serum at PN120. SC: saline control group receiving a commercial diet and gavage with saline (*n* = 10); SO: soybean oil group receiving a commercial diet and gavage with soybean oil (dose: 0.5 mL/100 g body mass) (*n* = 10); BURI: buriti oil group receiving a commercial diet and gavage with buriti oil (dose: 0.5 mL/100 g body mass) (*n* = 10); PN120: 120 days old. Results expressed as mean and standard error of the mean. Symbol * indicates statistical difference between groups SO and BURI versus SC (*p* < 0.05) (A). No symbol indicates no statistical difference among the groups (*p* > 0.05) (B–F).

The results related to serum redox balance are shown in Figure 3. At PN120, no significant differences were found in serum redox balance between groups in FRAP (Figure 3B), DPPH (Figure 3C), FOX (Figure 3D), TBARS (Figure 3E), and thiol (Figure 3F) analyses (*p* > 0.05). The BURI group showed a −20.6% reduction in carbonyl protein compared to the SC group (*p* < 0.05; Figure 5A), and the OS group reduced it by −22.7% (*p* < 0.05) compared to the SC group.

**FIGURE 4:**
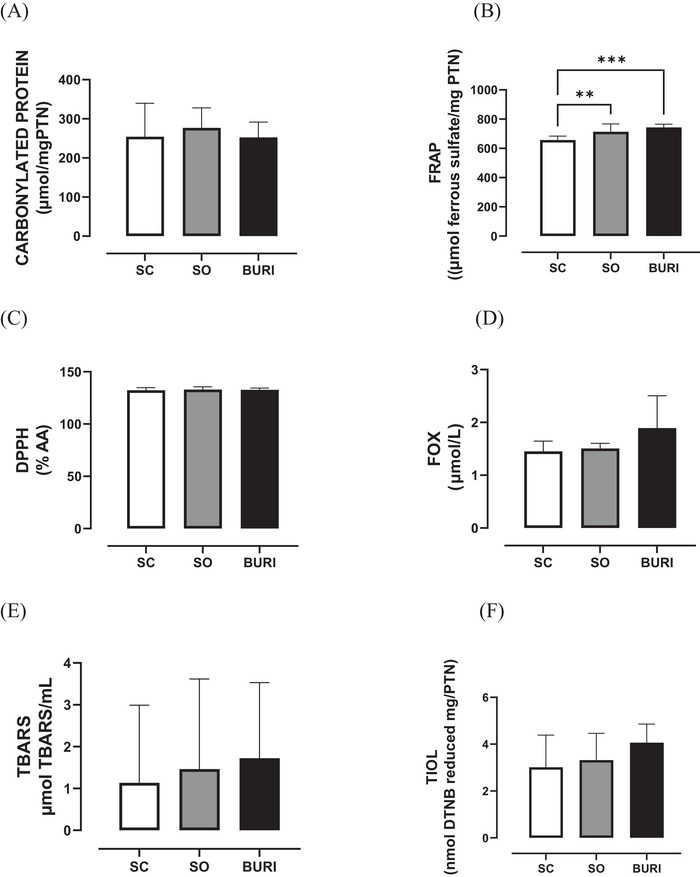
Hepatic redox balance of the animals at PN120. (A) Hepatic carbonylated protein (µmol mg/PTN) at PN120. (B) Hepatic FRAP (µmol ferrous sulfate/mg protein) at PN120. (C) Hepatic DPPH (mM DPPH/mg protein) at PN120. (D) Hepatic FOX (µmol/L) at PN120. (E) Hepatic TBARS (µmol TMP/mL) at PN120. (F) Thiol (nmol reduced DTNB/mg protein) hepatic at PN120. SC: saline control group receiving a commercial diet and gavage with saline (*n* = 10); SO: soybean oil group receiving a commercial diet and gavage with soybean oil (dose: 0.5 mL/100 g body mass) (*n* = 10); BURI: buriti oil group receiving a commercial diet and gavage with buriti oil (dose: 0.5 mL/100 g body mass) (*n* = 10); PN120: 120 days old. Results expressed as mean and standard error of the mean. Symbol ** indicates statistical difference between groups SO versus SC (*p* < 0.05) (B). Symbol *** indicates statistical difference between groups BURI versus SC (*p* < 0.05) (B). No symbol indicates no statistical difference among the groups (*p* > 0.05) (A, C, and D–F).

At PN120, no significant differences were observed in hepatic redox balance between groups in carbonyl protein (Figure 4A), DPPH (Figure 4C), FOX (Figure 4D), TBARS (Figure 4E), and thiol (Figure 4F) analyses (*p* > 0.05). In the FRAP analysis, the BURI group showed a +12.9% increase (*p* < 0.05, Figure 4B) and the SO group a +8.6% increase (*p* < 0.05; Figure 4B) when compared to the SC group. The results related to hepatic redox balance are shown in Figure 4.

The results related to the animals’ anthropometric parameters and tissue weights after supplementation are shown in Table 1. There were no differences in liver and adrenal weights among groups (*p* > 0.05). The BURI group showed a +27.7% increase in pancreas weight compared to the SC group (*p* < 0.05, Table 1). There were no significant differences among groups in white and brown adipose tissue weights, body mass index, Lee index, and nasoanal length (*p* > 0.05).

**TABLE 1:** Anthropometric parameters and body tissues in PN120.

	SC (*n* = 10)	SO (*n* = 10)	BURI (*n* = 10)	*p*‐value
Liver relative weight (g/100 g body mass)	3.148 ± 0.135^a^	2.85 ± 0.062^a^	2.854 ± 0.073^a^	>0.05
Pancreas (g/100 g body mass)	0.390 ± 0.014^a^	0.458 ± 0.029^ab^	0.499 ± 0.017^bc^	<0.05
Left adrenal (g/100 g body mass)	0.009 ± 0.001^a^	0.009 ± 0.000^a^	0.008 ± 0.000^a^	>0.05
Right adrenal (g/100 g body mass)	0.239 ± 0.031^a^	0.262 ± 0.027^a^	0.263 ± 0.0145^a^	>0.05
Retroperitoneal adipose tissue (g/100 g body mass)	1.835 ± 0.180^a^	1.544 ± 0.101^a^	1.558 ± 0.138^a^	>0.05
Epididymal adipose tissue (g/100 g body mass)	1.258 ± 0.098^a^	1.022 ± 0.041^a^	1.111 ± 0.092^a^	>0.05
Mesenteric adipose tissue (g/100 g body mass)	1.13 ± 0.109^a^	0.929 ± 0.084^a^	0.930 ± 0.092^a^	>0.05
Subcutaneous adipose tissue (g/100 g body mass)	0.107 ± 0.008^a^	0.102 ± 0.008^a^	0.109 ± 0.006^a^	>0.05
Brown adipose tissue (g/100 g body mass)	3.997 ± 0.404^a^	3.338 ± 0.156^a^	3.393 ± 0.265^a^	>0.05
BMI (g/cm^2^)	0.548 ± 0.019^a^	0.562 ± 0.009^a^	0.537 ± 0.007^a^	>0.05
Lee index (g/cm^3^)	7.069 ± 0.112^a^	7.035 ± 0.054^a^	7.024 ± 0.040^a^	>0.05
Nasoanal length (cm)	25.44 ± 0.242^a^	24.9 ± 0.179^a^	25.4 ± 0.163^a^	>0.05

*Note*: Results expressed as mean and standard error of the mean. Different subscript letters indicate statistical difference among groups (*p* < 0.05). SC: saline control group receiving a commercial diet and gavage with saline (*n* = 10); SO: soybean oil group receiving a commercial diet and gavage with soybean oil (dose: 0.5 mL/100 g body mass) (*n* = 10); BURI: buriti oil group receiving a commercial diet and gavage with buriti oil (dose: 0.5 mL/100 g body mass) (*n* = 10).

Abbreviation: BMI, body mass index.

The results obtained through biochemical parameter evaluations at PN120 are shown in Table 2 . At PN120, the BURI group showed a −33.3% reduction in triglycerides compared to the SC group (*p* < 0.05), as did the SO group, with a −37.1% decrease (*p* < 0.05). HDL decreased by −10.4% in the BURI group versus the SC group (*p* < 0.05). VLDL was −33.3% lower in the BURI group and −37.1% lower in the SO group, both compared to the SC group (*p* < 0.05). Total protein levels decreased by −8.4% in the BURI group and −7.2% in the SO group compared to the SC group (*p* < 0.05), and alanine aminotransferase was −32.6% lower in the BURI group when compared to the SC group (*p* < 0.05). Alkaline phosphatase was lower −34% in the BURI group and −26.8% in the SO group when compared to the SC group (*p* < 0.05). Calcium decreased by −7.9% in the BURI group and −7.7% in the OS group (*p* < 0.05). Iron was lower −32.7% in the BURI group and −22.2% in the OS group (*p* < 0.05). Magnesium decreased by −21.3% in the BURI group and −14.6% in the SO group (*p* < 0.05). Urea was −20.9% lower in the SO group, and uric acid was −30.9% lower in the BURI group, when compared to the SC group (*p* < 0.05). No significant differences were observed among groups in the biochemical parameters—total cholesterol, LDL‐c, albumin, oxaloacetic glutamic transaminase, direct and total bilirubin, phosphorus, creatinine, fasting glucose, serum insulin, and insulin resistance index among groups (*p* > 0.05) (Table 2).

**TABLE 2:** Serum biochemical parameters at PN120.

	SC	SO	BURI	*p*‐value
Triglycerides (mg/dL)	54.56 ± 5.116^a^	34.3 ± 2.578^b^	36.4 ± 3.631^b^	<0.05
Total cholesterol (mg/dL)	50.78 ± 1.899^a^	46 ± 2.62^a^	46.9 ± 1.616^a^	>0.05
HDL‐c (mg/dL)	24.44 ± 0.766^a^	22.3 ± 0.668^ab^	21.9 ± 0.690^b^	<0.05
VLDL‐c (mg/dL)	10.91 ± 1.023^a^	6.86 ± 0.516^b^	7.28 ± 0.726^b^	<0.05
LDL‐c (mg/dL)	15.42 ± 1.713^a^	16.84 ± 2.089^a^	17.72 ± 1.232^a^	>0.05
Albumin (g/dL)	3.256 ± 0.050^a^	3.14 ± 0.048^a^	3.12 ± 0.066^a^	>0.05
Total proteins (g/dL)	5.789 ± 0.122^a^	5.37 ± 0.109^b^	5.3 ± 0.116^b^	<0.05
Alanine aminotransferase (mg/dL)	50 ± 6.5^a^	39.9 ± 1.991^ab^	33.7 ± 1.66^b^	<0.05
Aspartate aminotransferase (mg/dL)	137 ± 9.625^a^	138.8 ± 8.701^a^	106.9 ± 11.92^a^	>0.05
Alkaline phosphatase (mg/dL)	86.89 ± 8.606^a^	63.6 ± 4.287^b^	57.3 ± 3.048^b^	<0.05
Direct bilirubin (mg/dL)	0.056 ± 0.006^a^	0.0566 ± 0.002^a^	0.042 ± 0.006^a^	>0.05
Total bilirubin (mg/dL)	0.11 ± 0.008^a^	0.13 ± 0.006^a^	0.109 ± 0.007^a^	>0.05
Phosphorus (g/dL)	6.833 ± 0.264^a^	6.25 ± 0.221^a^	6.54 ± 0.206^a^	>0.05
Calcium (mg/dL)	10.84 ± 0.300^a^	10 ± 0.104^b^	9.98 ± 0.18^b^	<0.05
Iron (µg/dL)	221.4 ± 11.64^a^	172.3 ± 9.179^b^	149 ± 4.607^b^	<0.05
Magnesium (g/dL)	3.011 ± 0.144^a^	2.57 ± 0.076^b^	2.37 ± 0.059^b^	<0.05
Urea (mg/dL)	58.56 ± 2.461^a^	46.3 ± 3.081^b^	51.2 ± 1.665^ab^	<0.05
Creatinine (mg/dL)	0.654 ± 0.015^a^	0.613 ± 0.018^a^	0.64 ± 0.010^a^	>0.05
Uric acid (mg/dL)	1.1 ± 0.093^a^	0.9 ± 0.067^ab^	0.76 ± 0.043^bc^	<0.05
Glycemia (mg/dL)	78.22 ± 3.349^a^	78 ± 2.49^a^	77.3 ± 1.342^a^	>0.05
Insulin (ng/mL)	2.28 ± 0.255^a^	1.77 ± 0.257^a^	1.81 ± 0.275^a^	>0.05
IRI (glycemia in mmol/dL)	238.5 ± 25.67^a^	174.1 ± 24.06^a^	228.8 ± 55.43^a^	>0.05

*Note*: Results expressed as mean and standard error of the mean. Different subscript letters indicate statistical difference among groups (*p* < 0.05). SC: saline control group receiving a commercial diet and gavage with saline (*n* = 10); SO: soybean oil group receiving a commercial diet and gavage with soybean oil (dose: 0.5 mL/100 g body mass) (*n* = 10); BURI: buriti oil group receiving a commercial diet and gavage with buriti oil (dose: 0.5 mL/100 g body mass) (*n* = 10).

Abbreviations: HDL‐c, high‐density lipoprotein cholesterol; IRI, insulin resistance index; LDL‐c, low‐density lipoprotein cholesterol; VLDL‐c, very‐low‐density lipoprotein cholesterol.

**TABLE 3:** Fatty acid composition in Buriti oil and soybean oil.

	Buriti oil		Soybean oil	
Fatty acid	Median (%)	SD	Median (%)	SD
Caprylic (C8:0)	ND	ND	ND	ND
Capric (C10:0)	ND	ND	ND	ND
Lauric (C12:0)	0.0230	0.0052	ND	ND
Mystic (C14:0)	0.0622	0.0037	0.0800	0.0009
Pentadecanoic (C15:0)	0.0392	0.0026	ND	ND
Palmitic (C16:0)	17.4887	0.5658	10.6600	0.0828
Palmitoleic acid (C16:1)	0.1484	0.0967	0.0800	0.0016
Heptadecanoic (C17:0)	0.0676	0.0031	0.0800	0.0019
Trans‐10‐heptadecenoic (C17:1)	0.0516	0.0013	0.0300	0.0393
Stearic (C18:0)	1.3024	0.1073	3.7000	0.0443
Oleic (C18:1 ω‐9)	78.2518	0.5779	28.4600	0.0249
Linoleic (C18:2)	1.6911	0.0118	50.6300	0.0044
α‐linolenic (C18:3 ω‐3)	0.7708	0.0049	5.3200	0.0041
Arachidic (C20:0)	0.0842	0.0016	0.3500	0.0227
Eicosadienoic (C20:2 ω‐6)	ND	ND	0.0800	0.0267
Eicosapentaenoic (C20:5 ω‐3)	0.1182	0.0021	0.0700	0.0387
Behenic (C22:0)	0.0097	0.0138	0.4300	0.0436
Tricosylic (C23:0)	0.0416	0.0057	0.0200	0.0306
Total	100.0		100.0	

Abbreviations: ND, not detected; SD, standard deviation.

**FIGURE 5:**
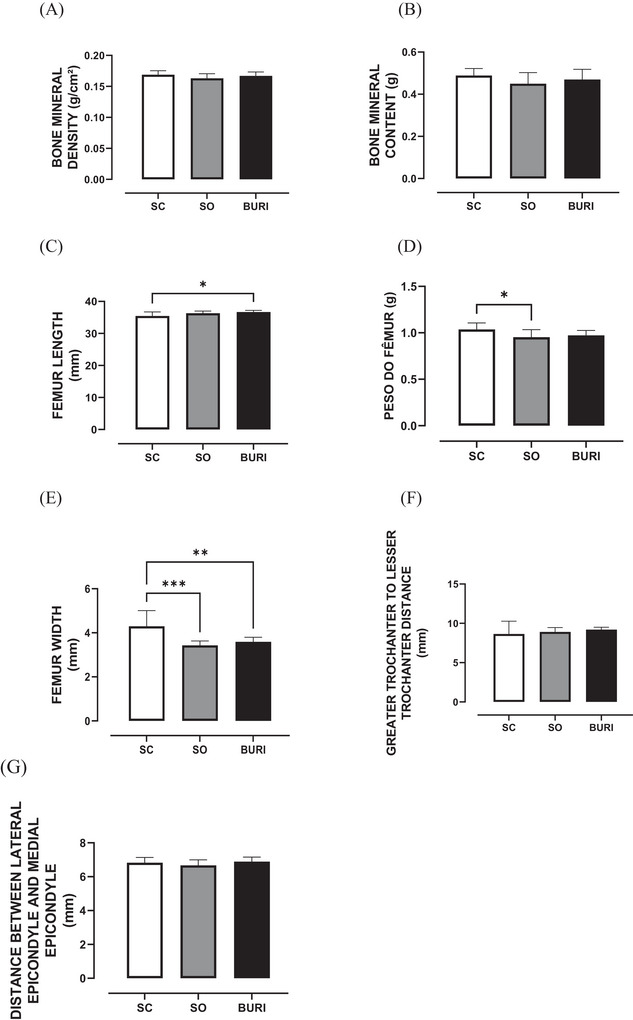
Anatomical parameters of the femur at PN120. (A) Bone mineral density (g/cm^2^) at PN120. (B) Bone mineral content (g) at PN120. (C) Femur length (mm) at PN120. (D) Femur weight (g) at PN120. (E) Femur width (mm) at PN120. (F) Distance between major trochanter and minor trochanter (mm) at PN120. (G) Distance between lateral epichondrium and medial epichondrium (mm) at PN120. SC: saline control group receiving a commercial diet and gavage with saline (*n* = 10); SO: soybean oil group receiving a commercial diet and gavage with soybean oil (dose: 0.5 mL/100 g body mass) (*n* = 10); BURI: buriti oil group receiving a commercial diet and gavage with buriti oil (dose: 0.5 mL/100 g body mass) (*n* = 10); PN120: 120 days old. Results expressed as mean and standard error of the mean. Symbol * indicates statistical difference between groups BURI versus SC (*p* < 0.05) (C). Symbol * indicates statistical difference between groups SO versus SC (*p* < 0.05) (D). Symbol *** indicates statistical difference between groups SO versus SC (*p* < 0.05) (E). Symbol ** indicates statistical difference between groups BURI versus SC (*p* < 0.05) (E). No symbol indicates no statistical difference among the groups (*p* > 0.05) (A, B, F, and G).

At PN120, no significant differences were observed between groups in femur anatomical parameters such as bone mineral density (Figure 5A), bone mineral content (Figure 5B), distance between greater and lesser trochanters (Figure 5F), and between lateral and medial epicondyles (Figure 5G) (*p* > 0.05). In femur anatomical parameter evaluation, the BURI group showed a +3.4% increase in length compared to SC (*p* < 0.05; Figure 5C). The OS group had an −8% reduction in weight (*p* < 0.05; Figure 5D). Regarding femur width, the BURI and SO groups showed reductions of −16.5% and −20.2%, respectively, compared to the SC group (*p* < 0.05; Figure 5E). The results related to femur anatomical parameters are shown in Figure 5.

**FIGURE 6:**
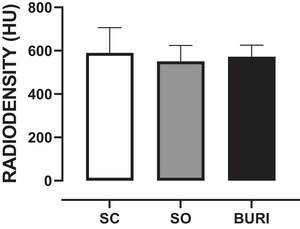
Radiodensity (HU) of the femoral head (tomography) at PN120. SC: saline control group receiving a commercial diet and gavage with saline solution (*n* = 10); SC: saline control group receiving a commercial diet and gavage with saline (*n* = 10); SO: soybean oil group receiving a commercial diet and gavage with soybean oil (dose: 0.5 mL/100 g body mass) (*n* = 10); BURI: buriti oil group receiving a commercial diet and gavage with buriti oil (dose: 0.5 mL/100 g body mass) (*n* = 10); PN120: 120 days old. Results expressed as mean and standard error of the mean. No symbol indicates no statistical difference among the groups (*p* > 0.05).

The results related to femoral head radiodensity are shown in Figure 6. At PN120, there was no significant difference in femoral head radiodensity among groups (Figure 6A) (*p* > 0.05).

**FIGURE 7:**
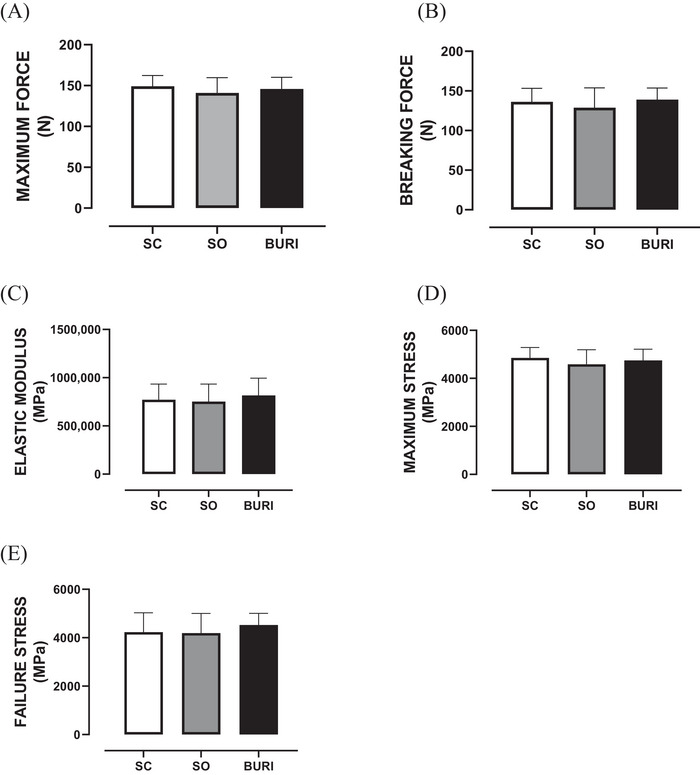
Biomechanical characteristics of the femur at PN120. (A) Maximum force (N) at PN120. (B) Breaking strength (N) at PN120. (C) Elastic modulus (MPa) at PN120. (D) Maximum voltage (N) at PN120. (E) Breakdown stress (MPa) at PN120. SC: saline control group receiving a commercial diet and gavage with saline (*n* = 10); SO: soybean oil group receiving a commercial diet and gavage with soybean oil (dose: 0.5 mL/100 g body mass) (*n* = 10); BURI: buriti oil group receiving a commercial diet and gavage with buriti oil (dose: 0.5 mL/100 g body mass) (*n* = 10); PN120: 120 days old. Results expressed as mean and standard error of the mean. No symbol indicates no statistical difference among the groups (*p* > 0.05).

At PN120, there were no significant differences among groups in femur biomechanical parameters such as maximum force (Figure 7A), breaking force (Figure 7B), elastic modulus (Figure 7C), maximum stress (Figure 7D), and breaking stress (Figure 7E) (*p* > 0.05). The results related to the biomechanical parameters of the femur are shown in Figure 7.

## 4 Discussion

This study evaluated the effects of 30 days of supplementation with buriti oil at a dose of 0.5 mL/100 g body weight. This dose was selected based on Raghu Nadhanan et al. (2013), who used the same volume of fish oil (0.5 mL/100 g body weight) and reported no adverse effects. Soybean oil was used as a secondary control because vegetable oils can promote satiety and potentially confound metabolic outcomes. Additionally, soybean oil is recommended as a lipid source for rodent diets by the American Institute of Nutrition (Reeves et al. 1993).

The dose used in the present study (0.5 mL/100 g body weight) corresponds to approximately 5 mL/kg in rats. Considering the density of buriti oil (0.91 g/mL), this is equivalent to 4.55 g/kg (4550 mg/kg) (de Andrade Mesquita et al. 2020). Using the body surface area conversion method proposed by Reagan‐Shaw et al. (2008), and applying *K*_m_ factors of 6 for rats and 37 for humans, the calculated human equivalent dose (HED) is approximately 738 mg/kg. For a 70‐kg adult, this corresponds to approximately 52 g/day or ∼57 mL/day (approximately four tablespoons).

In our experimental model, this dose corresponds to an estimated human intake of approximately 57 mL/day of oil, which exceeds the average consumption typically observed in non‐Mediterranean populations. In large US cohorts, olive oil intake is generally reported in the range of approximately 7–12 g/day (Guasch‐Ferré et al. 2020; Guasch‐Ferré et al. 2022; Tessier et al. 2024). In contrast, in traditional Mediterranean dietary contexts, particularly in Southern European populations, habitual olive oil consumption may reach approximately 25–50 mL/day, values ​​considered characteristic of a Mediterranean dietary pattern. Furthermore, recent Mediterranean cohort studies have stratified olive oil or extra virgin olive oil intake above 50 g/day, with these higher intake levels being associated with lower total, cardiovascular, and cancer‐related mortality (Farràs et al. 2021; Torres‐Collado et al. 2022; Bonfiglio et al. 2024). Although the dose used in this study exceeds typical human dietary intake levels, these findings provide evidence of the metabolic activity and safety profile of buriti oil supplementation in dose more elevated and support the rationale for further lower dose and human intervention studies.

From a nutritional perspective, buriti oil presents a lipid profile rich in monounsaturated fatty acids, particularly oleic acid, similar to olive oil, in addition to high levels of carotenoids and other bioactive compounds (Marcelino et al. 2022). These compositional characteristics suggest that buriti oil may confer health‐promoting effects consistent with those observed in dietary patterns rich in monounsaturated plant oils (Lotfi et al. 2021). In this context, buriti oil may represent a potentially health‐promoting lipid source and a complementary alternative to traditionally consumed edible oils, particularly in regions where *M. flexuosa* is native and culturally consumed (Dos Santos et al. 2015; Souto et al. 2025; De Almeida Sant Anna Trindade et al. 2025). However, the present findings should not be interpreted as evidence of direct substitution without further clinical and dietary intervention studies.

From a broader food system perspective, diversification of edible oil sources using native species such as buriti may contribute to reducing dependence on large‐scale monoculture‐based oil production systems. The incorporation of regionally adapted plant resources has the potential to support biodiversity conservation, local production chains, and food system resilience (Murphy 2025; Souto et al. 2025; De Almeida Sant Anna Trindade et al. 2025). However, sustainability implications require dedicated environmental, agronomic, and life‐cycle assessment studies and cannot be inferred solely from nutritional or biochemical outcomes.

Buriti oil supplementation led to significant changes, including reduced body mass and lower serum levels of carbonylated protein, triglycerides, HDL, VLDL, total proteins, ALT, alkaline phosphatase, calcium, iron, magnesium, and uric acid. Femur width decreased, while hepatic antioxidant activity (FRAP), pancreas weight, and femur length increased. Soybean oil supplementation reduced serum levels of carbonylated protein, triglycerides, VLDL, total proteins, alkaline phosphatase, calcium, iron, and urea, and decreased femur weight and width compared to SC.

In this study, buriti oil supplementation significantly reduced body mass. However, Aquino et al. (2015) found no such effect in 30 Wistar rats supplemented for 28 days with crude or refined oil (7 g/100 g diet). Similarly, Marcelino et al. (2022) observed no changes in weight gain or food intake in Swiss mice gavage with buriti (dose: 1000 and 2000 mg/kg) or extra virgin olive oil (dose: 1000 and 2000 mg/kg) for 90 days. Despite differences between studies regarding species, duration of intervention, dose, and route of administration, these data suggest that the effects of buriti oil on body mass may be dependent on the experimental context.

In this sense, the reduction in body mass observed in the present study may be related to the composition of buriti oil, rich in oleic acid, carotenoids, and antioxidant compounds, which have been associated with the modulation of lipid metabolism and increased fatty acid oxidation (Reis et al. 2020; De Souza Aquino et al. 2023). Regarding food intake, it remained stable between the groups, corroborating the findings of Aquino et al. (2015), indicating that the observed effect does not result from reduced satiety, but possibly from metabolic effects, which reinforces the potential of buriti oil as a functional ingredient in specific nutritional strategies.

This study found no significant differences in body composition among groups, including fat and lean mass, or bone parameters (area, content, mineral density). However, literature reports vary with different dietary fats. Lac et al. (2008) found that a commercial diet, with 15 g of commercial vegetable oil during 10 weeks, reduced body and lean mass and bone parameters in Wistar male rats. Ferolla Da Camara Boueri et al. (2015) reported similar effects with flaxseed oil until PN60, when weaned rats received a purified diet added with 7 g of flaxseed oil for 39 days. On the other hand, flaxseed oil gavage (dose: 0.5 mL/100 g body mass) to dams during lactation (21 days consecutively) showed a reduction in body fat mass in offspring at weaning and higher visceral fat mass at adulthood in male rats (Guarda et al. 2014; Guarda et al. 2016). These discrepancies may be related to multiple factors, including the type of oil, the lipid ratio of the diet, the route of administration, the age of the animals, the duration of the intervention, and the presence of specific bioactive compounds.

In this context, it is noteworthy that buriti oil has a higher content of monounsaturated fatty acids, in contrast to flaxseed oil, which is characterized by a high content of polyunsaturated fatty acids, which may result in distinct metabolic responses (de Andrade Mesquita et al. 2020; Deme et al. 2021; Hua et al. 2022). Furthermore, to date, no studies have been identified that evaluate the effects of buriti oil supplementation on body composition using DXA, reinforcing the novel nature of the approach adopted in this study.

Although DPPH, FOX, T‐BARS, and thiol markers showed no significant changes, serum carbonylated protein decreased and hepatic FRAP increased in groups supplemented with buriti and soybean oils, suggesting an antioxidant effect and reduced protein peroxidation. Similarly, Nunes Lage et al. (2018) found that a purified diet containing 2% buriti pulp flour, offered to female Fischer diabetic rats for 30 days, reduced liver and heart carbonylation in diabetic rats, supporting our findings and the hypothesis that buriti helps to regulate oxidative stress. Méndez et al. (2013) reported that female Wistar rats that received a weekly oral dose of 0.8 mL/kg of fish oil (EPA and DHA) for 13 weeks showed reduced protein oxidation in serum, muscle, and liver. Walczewska et al. (2010), in a study, evaluated low‐fat diets (10% energy from fat) and high‐fat diets (40% energy from fat) enriched with lard, sunflower oil, or fish oil, and found that male Wistar rats that received a high‐fat diet enriched with sunflower oil showed increased plasma FRAP but also increased cellular stress, while rats that received a high‐fat diet enriched with fish oil showed improved redox status without promoting intracellular oxidation. De Souza Aquino et al. (2023) observed increased hepatic glutathione peroxidase activity in rats receiving buriti oil under iron overload. The FRAP increase may be linked to bioactive compounds in buriti oil, especially carotenoids and polyphenols (Amorim et al. 2021). Beta‐carotene and tocopherols, present in buriti oil, are potent antioxidants capable of quenching reactive oxygen species, which may contribute to the reduced carbonylated protein levels observed in the BURI group, as suggested for carotenoids by Saini et al. (2022). Overall, our results and the literature suggest that buriti oil acts as a serum and hepatic antioxidant, with lower protein carbonylation and higher FRAP in the BURI and SO groups, indicating protective effects on redox balance and potential nutritional and therapeutic applications.

Tissue analysis showed no significant differences in liver or adrenal weights, indicating that buriti oil did not cause structural changes. White and brown fat were also unaffected, suggesting no impact on fat accumulation. Marcelino et al. (2022) reported similar results after gavage with buriti oil (dose: 1000 and 2000 mg/kg) for 90 days of supplementation. Conversely, De Souza Aquino et al. (2023) demonstrated that Wistar rats that received a high daily oral dose of FeSO_4_ (60 mg/kg body weight) and were fed a diet containing either soybean or buriti oil for 17 days under iron overload showed reduced liver weight when fed the buriti oil diet compared with rats with iron overload fed the soybean oil diet, suggesting a protective effect of buriti against oxidative stress. Guarda et al. (2016) showed that the effects of vegetable oil vary with lipid composition and intake duration, in which dams received flaxseed oil or soybean oil by gavage (dose: 0.5 mL/100 g body mass) during lactation (21 days consecutively), and the offspring presented higher visceral and total fat mass in adulthood, despite no changes in liver and body weights. In contrast, another study in rats with iron overload that received a diet enriched with buriti oil did not show changes in body composition or organ weights, which suggests a neutral effect in healthy models without toxicity, but may be an important nutrition strategy under metabolic stress, such as obesity or diabetes (De Souza Aquino et al. 2023).

A significant increase in relative pancreatic weight was observed in the BURI group compared to the control group, possibly reflecting adaptation to a lipid‐rich diet and buriti oil antioxidants. However, an inflammatory or compensatory effect could not be discarded, as Laget et al. (2021) demonstrated that male Wistar rats that received a high‐fat diet (56% energy from fat) enriched with soybean oil (2.5%), palm oil (30%), or lard for 12 weeks showed adipocyte hypertrophy and macrophage infiltration after palm oil or lard intake. Roche et al. (2013), in a study, replaced the amount of lipid in a purified diet with virgin olive oil, sunflower oil (SOF), or fish oil and offered it for 2 months to male Wistar rats, and showed reduced pancreatic damage in aged rats receiving olive oil, highlighting the role of lipid profile. Despite buriti's protective bioactive compounds, we observed in our study higher pancreas weight in male rats that received gavage with buriti oil. Thus, we suggested that histological and inflammatory analyses are needed to clarify this response.

Similarly, Aquino et al. (2016) found no differences in BMI or thoracic and abdominal circumferences in Wistar rats fed biscuits with buriti oil for 28 days. Aquino et al. (2015) also reported no changes in BMI, Lee index, or body length after crude or refined buriti oil supplementation. These findings support the present study and suggest that buriti oil, regardless of form, does not impair growth or body composition, reinforcing its potential as a nutritional source of bioactive compounds.

Buriti and soybean oil supplementation significantly reduced triglycerides and VLDL, likely due to antioxidants and unsaturated fats. However, HDL decreased in the BURI group, as also reported by Marcelino et al. (2022). Aquino et al. (2015) found reductions in triglycerides, HDL, VLDL, total cholesterol, and LDL in young rats. These findings suggest that buriti oil's effects on lipid profile depend on species, dose, duration, and health status. Despite the HDL reduction, its metabolic benefits are promising, and further studies are needed, especially in dyslipidemic models. These lipid‐modulating effects may be partly related to the oil's high oleic acid content, as monounsaturated fatty acids are known to promote healthier blood lipid profiles (Gillingham et al. 2011).

In our study, we demonstrated that buriti oil gavage reduced serum and alkaline phosphatase, which suggested an effect of buriti oil on liver metabolism, despite unchanged AST in the BURI group. On the other hand, De Souza Aquino et al. (2023) demonstrated that Wistar rats that received a high daily oral dose of FeSO4 and were fed a diet containing buriti oil for 17 days under iron overload showed changes in hepatic enzymes. However, Aquino et al. (2015) demonstrated that a diet containing soybean oil (control), crude buriti oil, or refined buriti oil (7 g oil/100 g diet for growing rodents), when offered to male Wistar rats for 28 days, resulted in increased AST levels with crude buriti oil. These findings suggested that hepatic effects may depend on refinement and administration, warranting further investigation.

In our study, we observed a reduction in serum urea and uric acid, which may reflect an improvement in renal function or less protein degradation. Thus, the serum uric acid can be directly related to the risk of kidney diseases, and this decrease suggests renal protection. In the same direction, a study demonstrated that supplementation with extra virgin oil and its fraction for 4 weeks in male Wistar rats induced biochemical changes in the kidney, accompanied by a significant decrease in lipid peroxidation and an increase in the level of the antioxidant defense system (Nakbi et al. 2012).

In this study, supplementation with soybean and buriti oil significantly reduced serum iron, suggesting effects on mineral metabolism. De Souza Aquino et al. (2023) found similar results in iron‐overloaded rats, where the oil was able to reduce serum iron concentrations in these rats, mitigating the damage resulting from the overload through antioxidants present in buriti oil. In this case, the reduction occurred in healthy animals supplemented with soybean oil and buriti oil, suggesting possible interference in the mechanisms of absorption, transport, and mineral metabolism associated with the lipid matrix of these oils.

The observed reduction in serum iron levels following buriti oil supplementation warrants careful consideration; although no clinical signs of anemia were observed, this finding may suggest potential interference with iron absorption or metabolism. Beta‐carotene and oleic acid present in buriti oil can be shown to form complexes with iron, potentially reducing its bioavailability. This reduction could reflect redistribution of iron to tissues or modulation of hepcidin expression, which regulates systemic iron homeostasis (Ganz and Nemeth 2012; De Souza Aquino et al. 2023). Furthermore, the reduction in serum iron levels may be associated with the greater hepatic antioxidant protection observed, since decreased iron availability can limit the formation of iron‐mediated reactive oxygen species and, consequently, reduce hepatic oxidative stress (Kawabata 2022; Gensluckner et al. 2024). Long‐term studies with assessment of hematological parameters, tissue iron stores, and iron status are needed to clarify the potential implications of this finding.

Magnesium and calcium levels also decreased, indicating a broader mineral modulation. Along the same lines, similar effects were observed in a study with ovariectomized rats supplemented with extra virgin olive oil (Azeite‐OVX) orally for 12 weeks (Saleh and Saleh 2011). Ali Bakr and Saad Alyam (2023) demonstrated that male albino rats with paracetamol liver intoxication that received an oral dose (1 mL/kg) of *Nigella sativa* black and *Lactuca sativa* oils showed a significant decrease in unsaturated iron binding capacity (UIBC), creatine kinase (CK), magnesium (Mg), phosphorus (Phos.), and iron (Fe), compared to rats with liver intoxication (Ali Bakr and Saad Alyam 2023). Although these studies were conducted in different pathophysiological contexts, the consistency among the results suggests that the dietary lipid matrix may influence mechanisms related to intestinal absorption, transport, and systemic redistribution of minerals (Corte‐Real and Bohn 2018).

In the present study, these changes occurred in healthy animals and over a shorter experimental period, suggesting an adaptive metabolic adjustment, rather than a deleterious effect. The interaction between monounsaturated fatty acids, antioxidant compounds, and the formation of intestinal micelles can modify the bioavailability of minerals such as magnesium and calcium, in addition to influencing hepatic regulatory pathways involved in energy and mineral metabolism (Rishi and Subramaniam 2017; Corte‐Real and Bohn 2018; Rolić et al. 2025). Thus, the data indicate that oils rich in monounsaturated fatty acids, such as buriti oil, can subtly modulate mineral homeostasis, highlighting the importance of future investigations that evaluate different exposure times, physiological states, and tissue distribution of these minerals for a better understanding of their metabolic implications.

On the other hand, no significant differences were found in fasting glucose, insulin, or insulin resistance, which suggested that buriti oil did not affect glycemic control. Our findings indicate that buriti oil does not alter carbohydrate metabolism under the tested conditions. Nunes Lage et al. (2018) and Aquino et al. (2016) demonstrated that a diet containing 2% buriti pulp flour or biscuits with buriti oil, offered to an animal model for approximately 30 days, did not present changes in glucose metabolism. Another study with aged rats, which replaced the amount of lipid in a purified diet for virgin olive oil, sunflower oil (SOF), or fish oil and offered for 2 months to male Wistar rats, observed that olive oil had no impact on glucose metabolism; however, sunflower oil caused hyperinsulinemia, and fish oil increased TNF‐α and pancreatic fibrosis (Roche et al. 2013). Thus, our findings are the first study to demonstrate the effects of buriti oil supplementation on serum insulin concentrations and insulin resistance. Thus, further research is needed with varying doses and durations.

In our study, buriti oil supplementation for 30 days reduced femur width but increased femur length, which suggests structural bone changes, followed by lower serum calcium. On the other hand, Pereira et al. (2021) demonstrated that in Wistar rats, offspring that received a diet containing 7% of flaxseed oil from birth to 67 days exhibited higher femur width, weight, bone mineral density, head radiodensity, maximum force, breaking strength, and cortical thickness. In the same direction, Chen et al. (2020) demonstrated that in male Sprague–Dawley rats fed a high‐fat diet containing 10% flaxseed oil for 22 weeks, flaxseed oil improved bone parameters such as trabecular volumetric bone mineral density, trabecular bone volume/total volume, trabecular number, and trabecular thickness, compared with the high‐fat diet group. These findings suggest that vegetable oils may differentially influence bone, depending on their lipid profile. Furthermore, alterations in bone metabolism may be associated with changes in relevant biochemical parameters, such as the availability of circulating minerals, including serum calcium (Hernández‐Becerra et al. 2017). Thus, more studies are necessary to elucidate the molecular mechanisms involved in the regulation of bone metabolism and proteins related to osteogenesis.

Thereby, in our study, no differences in femoral head radiodensity were found, which indicated that buriti oil did not affect bone mineral density. However, Pereira et al. (2021) reported increased radiodensity with flaxseed oil added to a diet offered to Wistar rats. Other studies with ovariectomized rats analyzed the effects of extra virgin olive oil (100 µL or 200 µL/day by oral gavage for 3 months) and camelina oil (5 g or 9 g/kg body weight for 6 weeks) on bone health and demonstrated that both oils improved bone parameters (Díaz‐Curiel et al. 2020; Puzio et al. 2021). These findings suggest that monounsaturated fatty acid‐rich oils may prevent bone loss, especially under hormonal deficiency. However, our study with buriti oil showed no effect on bone parameters. Thus, more studies are necessary to relate the dosage, treatment duration, and molecular mechanisms involved with bone tissue.

In our study, biomechanical femur analysis showed no significant differences, indicating buriti oil supplementation did not affect bone strength. Maximum force, fracture force, and elastic modulus remained stable. Zakaria et al. (2017) demonstrated, in an alcohol experimental model with Sprague–Dawley male rats, that supplementation with palm vitamin E was able to improve bone biomechanical parameters, such as maximum force, ultimate stress, and Young's modulus.

Another study with male and female mice fed either a 10% flaxseed oil (FO) or a 10% corn oil (CO) diet from postnatal day (PND) 28 until PND 91 demonstrated that flaxseed oil did not change bone strength in growing mice (Cohen and Ward 2005). Thus, effects on bone resistance vary by oil type, model, and conditions; buriti oil showed a similar neutral profile in our study.

Notably, no in vivo studies have yet evaluated buriti oil's effects on bone health, leaving its impact largely unexplored. Thus, this study advances knowledge by addressing this gap.

Although the results are promising, the sample size and use of only healthy male rats may limit broader conclusions, but buriti oil did not present toxicity at the dose we used. However, the 30‐day period may also not reflect long‐term effects. In summary, although there are signs of biochemical and physiological benefits, the effects of buriti oil appear to depend on complex metabolic interactions. The lack of changes in some variables, alongside specific alterations, suggests targeted mechanisms, possibly involving oxidative stress modulation, lipid metabolism, and mineral balance.

We emphasize that the primary objective of the study was rather to evaluate the metabolic safety profile and biological responses to a defined exposure of buriti oil under controlled experimental conditions. Although the absence of significant changes in several analyzed parameters at this exposure level corroborates the interpretation that buriti oil did not induce evident adverse metabolic, hepatic, or renal effects in healthy animals, suggesting a metabolic safety profile under the evaluated conditions, these findings should be interpreted with caution. In metabolically healthy models, the maintenance of parameters within normal limits may reflect the preservation of metabolic stability and systemic homeostasis, as well as adaptive physiological responses to the intervention. The short duration of the intervention, associated with the use of a single dose, may have limited the detection of more subtle metabolic effects; thus, the absence of statistical differences does not exclude the occurrence of physiological adaptations or effects dependent on longer exposure times or different metabolic contexts.

## 5 Conclusion

Buriti oil stands out for its high content of bioactive compounds such as carotenoids, tocopherols, phytosterols, and mono‐ and polyunsaturated fatty acids, providing antioxidant and anti‐inflammatory properties of nutritional significance.

The results indicate that buriti oil supplementation improved the lipid profile as well as several biochemical parameters, including hepatic markers and anthropometric measures. Although some variables did not reach statistical significance, a marked reduction in body mass was observed in the buriti oil group, without evidence of toxicity. However, buriti oil intake was associated with a reduction in key minerals related to metabolism and bone health. Additionally, buriti oil showed promising effects on serum and hepatic redox balance, likely due to its high content of bioactive compounds. Therefore, buriti oil may represent a novel nutritional strategy and a potential ingredient for the development of new food products, contributing to improved dietary intake and increased economic value. Therefore, the observed reductions in serum minerals may reflect physiological configurations in mineral homeostasis, associated with the interaction between the dietary lipid matrix and not necessarily an exclusion effect or mineral deficiency. It is important to emphasize that the effects observed in the present study were obtained using a relatively high experimental dose, exceeding typical human dietary intake, and therefore should be interpreted as evidence of biological activity and safety, and not as a direct dietary recommendation. Therefore, further studies are needed to determine safe doses for human consumption and to elucidate the molecular mechanisms involved in metabolic regulation.

## Author Contributions

**Letícia de Almeida Sant’ Anna Trindade**: conceptualization, investigation, methodology, writing – original draft, formal analysis, data curation, writing – review and editing. **Beatriz Alem Nascimento de Araújo**: conceptualization, investigation, methodology. **Gabriel de Alcantara Noblat**: methodology, investigation. **Karen Pereira Coutinho**: investigation, methodology, data curation. **Karla de Araújo Coutinho**: data curation, methodology, investigation. **Renata Nascimento Matoso Souto**: methodology. **Anderson Junger Teodoro**: methodology, visualization, data curation, resources. **Aline D'Avila Pereira**: software, formal analysis, visualization, validation, methodology. **Mariana Sarto Figueiredo**: conceptualization, investigation, funding acquisition, visualization, validation, methodology, software, formal analysis, project administration, supervision, resources, data curation, writing – original draft, writing – review and editing.

## Funding

This work was supported by Fundação de Amparo à Pesquisa do Estado do Rio de Janeiro (FAPERJ, E26/211.451/2021).

## Conflicts of Interest

The authors declare no conflicts of interest.
